# Spontaneous Expulsion of Rectally Inserted Tramadol Ampoules: An Unusual Case Report of Drug Concealment and Delayed Disclosure

**DOI:** 10.1155/cris/5630002

**Published:** 2026-06-10

**Authors:** Ashish Sapkota, Priyanka Regmi, Ajay Kumar Yadav, Rajani Giri

**Affiliations:** ^1^ Department of General Practice and Emergency Medicine, BP Koirala Institute of Health Sciences (BPKIHS), Dharan, Nepal, bpkihs.edu

**Keywords:** ampoules, body stuffers, foreign body, rectum, spontaneous expulsion

## Abstract

Concealment of illicit drug ampoules is an unusual clinical scenario, posing diagnostic and management challenges due to delayed disclosure, drug leakage risk, and retrieval difficulties. We present the case of a 28‐year‐old male, an intravenous drug user, brought from police custody with complaints of per rectal pain. After initial denial, he admitted to rectal insertion of six tramadol ampoules. Clinical examination and imaging confirmed the presence of foreign bodies in the rectum. While surgical removal was planned, spontaneous expulsion occurred without complications, as verified by postoperative imaging. This case underscores the importance of early suspicion, detailed history‐taking, and prompt imaging in suspected rectal foreign bodies. In stable patients, conservative management with laxatives may allow safe natural expulsion, reducing the need for invasive intervention, as in this case; however, this should be seen as a positive outcome rather than a recommended management approach, especially in cases involving fragile drug‐containing ampoules.

## 1. Introduction

Descriptions of rectal foreign body insertion appear sporadically in the surgical literature, with historical reports dating back as far as the 16th century [1]. The motives for such insertions vary, commonly including autoerotic practices, concealment, attention‐seeking, accidental placement, assault, and occasionally attempts at relieving constipation [2]. Reports from Asian countries are relatively uncommon, whereas a larger body of literature originates from Eastern Europe [3]. These events occur more often in urban populations, with men in their third and fourth decades comprising the majority of affected patients. In emergency settings, individuals are frequently reluctant to provide accurate histories, which can complicate evaluation and delay definitive care [4, 5].

Removal of an intrarectal foreign object is a complicated issue for surgeons. Locating and extracting the item must be approached as an urgent intervention, given the potential for serious complications [6] Furthermore, inserting drug ampules into the rectum for “body‐packing” is a rare occurrence. It is primarily used to transport heroin, cocaine, amphetamines, and cannabis [7]. Individuals who engage in body packing typically arrive at the emergency department due to drug toxicity, intestinal obstruction, or, more frequently, at the request of law enforcement for medical verification or to rule out the possibility of body packing [8].

Here, we present a case of foreign body insertion of tramadol ampoules and concealment of truth, leading to unwarranted therapeutic intervention. This case report has been reported in line with the SCARE criteria [9].

## 2. Case Presentation

A 28‐year‐old married male, known intravenous drug user, presented to the Emergency Medicine Department from police custody with a complaint of per rectal pain for a day. In the primary survey, the patient appeared anxious with a patent and clear airway with oxygen saturation of 97% in room air and a respiratory rate of 22 breaths a minute. Pulse measured 102 beats a minute and blood pressure of 140/90 mmHg. The patient was alert and oriented (AVPU: alert). The patient was admitted, and a detailed history and secondary survey were done.

Law enforcement officers apprehended the patient on suspicion of drug trafficking. It was alleged that he had concealed multiple drug ampoules internally, a common practice known as “body stuffing.” During initial police interrogation, the patient vehemently denied any such activity, claiming no ingestion or rectal insertion of contraband. Approximately 24 h after his detention, he began complaining of intense pain in the rectal area, described as deep and throbbing in character, exacerbated on walking, and severe enough to make walking difficult. Concerned by the symptoms, he finally admitted to inserting six ampoules of tramadol rectally just before his arrest, and then he was rushed to the emergency department. The patient then added that he had passed one intact ampoule in his feces earlier that morning but claimed the remaining five were still in place. Despite direct questioning, he refused to give clear details about the timeline or whether any additional ampoules had been expelled. He denied any sudden onset of confusion, drowsiness, seizures, respiratory depression, tachycardia, palpitation, diaphoresis, etc. He also added his personal history of regular smoking and alcohol intake and occasional use of IV drugs.

On examination, the impression of foreign bodies in the rectum was felt in digital rectal examination with other systemic examination and detailed general head‐to‐toe examination revealing no significant findings. Initial management was started. An intravenous line was secured, and tramadol 50 mg IV for pain relief, ceftriaxone 1 gm IV prophylactically, and syrup lactulose 30 mL were started on stat. Then, the patient was closely monitored for signs of opioid toxicity, including respiratory depression and altered mental status.

**Figure 1:**
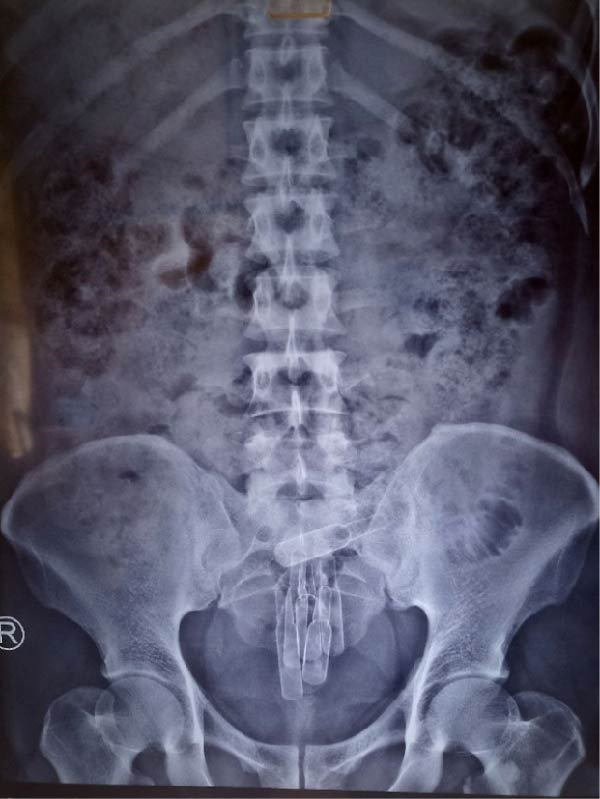
X‐ray abdomen + pelvis AP view revealing radio‐opaque ampoules in rectum with multiple air‐fluid levels in the large bowel.

**Figure 2:**
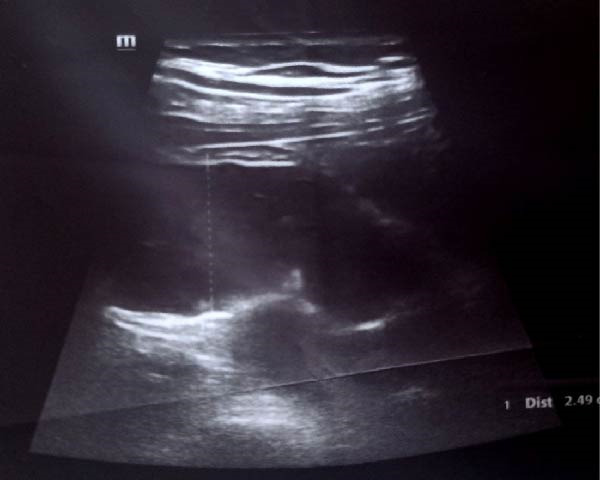
USG abdomen + pelvis revealing a few prominent small bowel loops.

**Figure 3:**
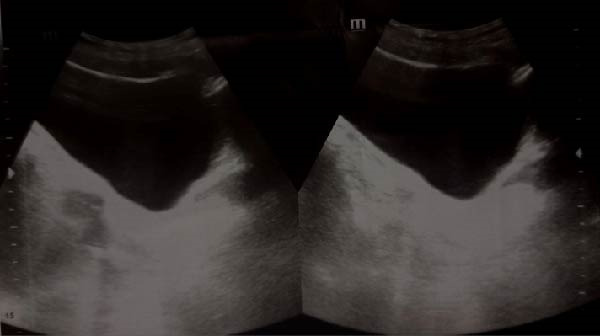
USG abdomen + pelvis showing a few tubular hyperechoic structures in the rectum, giving the impression of foreign bodies.

Then, the patient was advised to undergo hematological evaluation for infections or systemic effects and imaging. An X‐ray AP supine view of abdomen + pelvis revealed linear radiopaque densities in the pelvic cavity at the level of rectum, consistent with glass ampoules along dilated loops of large bowel with air‐fluid levels as shown in Figure 1. For further evaluation, a USG abdomen and pelvis was done, which revealed hyperechoic linear structures with posterior acoustic shadowing in the pelvis/rectal area—consistent with a foreign body in the rectum, along with the surrounding bowel loops appearing distended and fluid‐filled, which may correlate with bowel obstruction as shown in Figures 2 and 3. Complete hemogram and biochemical investigation were normal; however, the serology report showed a reactivity for HCV.

**Figure 4:**
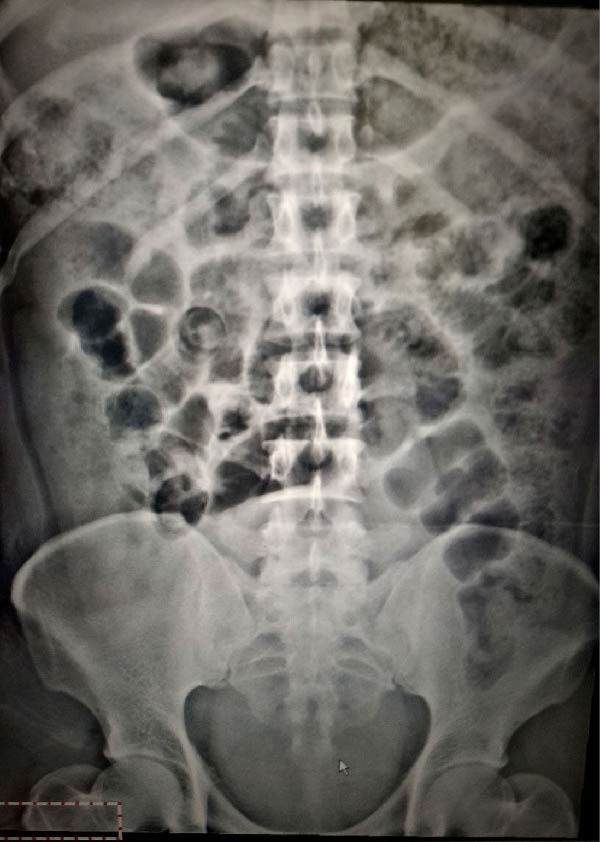
X‐ray abdomen + pelvis AP view revealing a few prominent bowel loops filled with gas and rectum devoid of any foreign bodies.

He was counseled on the need for surgical consultation and exploration under anesthesia. After appropriate discussion with the surgical colleagues, he was transferred to the emergency operating theatre, a spinal block was given with bupivacaine 1.2 mL (0.5%), and surgical exploration with proctoscopy and foreign body removal was planned. To the clinical team’s surprise, no ampoules were found in the rectum or distal colon. The mucosa appeared intact with no signs of trauma or perforation. A repeat emergency X‐ray supine AP of abdomen and pelvis was performed postprocedure, and this time, no foreign bodies were visible as in Figure 4, confirming that the ampoules had likely been passed spontaneously and unnoticed.

Under further interrogation, the patient admitted to possibly passing more ampoules during defecation but concealed this to avoid legal implications. The patient was discharged on the fourth day in stable condition after tolerating a normal diet and passing stools without difficulty. He received counseling about the risks of rectal foreign body insertion and the legal consequences of hiding relevant medical history. No postoperative or postanesthetic complications were observed. He was advised to avoid such practices and to seek medical help early if symptoms return. Follow‐up at 2 weeks and 1 month showed no signs of anorectal injury, infection, or recurrence.

## 3. Discussion

The retrieval of rectal foreign bodies poses significant technical challenges, largely due to the wide range of objects used and the anatomical complexity of the rectosigmoid junction. Sharp angulation and limited access can hinder straightforward extraction and may allow objects to migrate proximally into the sigmoid colon, further complicating management [10]. Demographically, there is a bimodal pattern of presentation; younger individuals in their twenties often present following autoerotic or coerced insertion, while older individuals in their sixties mainly present for prostatic massage and breaking fecal impactions [3]. Reported objects include glass and plastic bottles, cosmetic containers, wooden implements, and various improvised household items [11]. The most frequent underlying cause, however, remains sexual gratification [12]. Rectal foreign bodies are also common in the context of drug smuggling, known as body packing [5].

Drug concealment within the body is classified into three entities: body packing, body stuffing, and body pushing. Body packing involves the planned ingestion or insertion of well‐packaged drug packets, usually wrapped in multiple layers to prevent leakage. In contrast, body stuffing refers to the hurried concealment of poorly wrapped substances, often during imminent arrest, carrying a higher risk of rupture and toxicity. Body pushing describes the insertion of drug‐containing objects into body orifices such as the rectum or vagina, typically involving fewer items and minimal protective packaging [13, 14]. The present case does not conform to classical body packing, as the tramadol ampoules were inserted rectally without any protective wrapping. Instead, it more closely resembles body pushing with features of body stuffing, given the absence of packaging and the high risk of rupture. This distinction is clinically important, as unprotected drug‐containing objects, particularly fragile glass ampoules, carry a substantially higher risk of mechanical injury, leakage, and acute toxicity compared to well‐packaged drug packets described in classical body packing. A similar report by Giri et al. [7] described a patient who landed up in emergency with five ampoules of diazepam inserted per rectum for body packing with subsequent rectal bleeding after one ampoule broke in an attempt to extract the lowest lying ampoule.

The clinical presentation can differ among individuals; however, patients generally report experiencing rectal or abdominal pain, constipation, obstipation, bright red blood from the rectum, or incontinence. In cases where patients have a bowel perforation, various signs and symptoms may manifest, including significant guarding, rebound tenderness, and fever. Additionally, these patients may exhibit signs of septic shock [5]. A careful abdominal and digital rectal examination remains the cornerstone of evaluation, supplemented by radiography to confirm the object’s presence and location [15]. In our case, the patient complained only of rectal pain and difficulty in ambulation, without rectal bleeding, obstructive symptoms, or systemic signs of tramadol toxicity, suggesting intact ampoules without rupture or leakage. The digital rectal examination and X‐ray AP supine view of abdomen + pelvis done in the initial assessment of the case were consistent with the findings of foreign body impaction with drug ampoules in the rectum. Although computed tomography (CT) can provide valuable information regarding bowel wall integrity, perforation, or surrounding inflammation, it was not performed in this case due to the patient’s stable clinical condition, absence of signs suggestive of perforation or peritonitis, and the limitations in the resources.

Rectal foreign bodies are commonly categorized as high‐lying or low‐lying based on their position relative to the rectosigmoid junction. This distinction is crucial for management, as palpable, low‐lying objects are often removed transanally in the emergency department, whereas high‐lying objects frequently require surgical intervention [15, 16]. While transanal retrieval is preferred, laparotomy is indicated when transanal or endoscopic removal fails or if complications such as bowel perforation occur [17]. The use of laxatives is typically discouraged due to limited efficacy and the potential increased risk of injury to the bowel [16].

However, one could argue that this method of clearance may allow the patient to avoid a general anesthetic, as well as the risk of iatrogenic injury secondary to prolonged and repeated instrumentation of the rectum [10, 18]. However, the use of laxatives in the management of rectal foreign bodies remains controversial and is generally discouraged, particularly in cases involving fragile or sharp objects. In the present case, spontaneous expulsion occurred without complications; however, this should be considered a fortunate outcome rather than a recommended approach. Importantly, a report by Giri et al. [7] described rectal insertion of diazepam ampoules complicated by ampoule rupture and subsequent rectal bleeding during attempted extraction. This finding underscores the significant risk associated with fragile glass ampoules, particularly the potential for mucosal injury, perforation, and systemic drug exposure following rupture, rather than supporting expectant or laxative‐assisted management. Given that the retained objects in our case were unprotected glass ampoules containing a pharmacologically active substance, the risk profile is inherently high. Although the patient in this case was hemodynamically stable and initially managed with analgesia and lactulose while preparing for definitive intervention, the outcome should not be generalized. Management decisions must be individualized and guided by careful risk assessment, with particular consideration of the size, shape, fragility, and contents of the foreign body.

Management of a foreign body in the rectum is often challenging for a surgeon due to the variation in time of insertion, associated injuries, the type and location of an object, and the variation in anatomy [19]. One of the greatest challenges in these scenarios is delayed presentation, as many patients are embarrassed and reluctant to seek medical care promptly [5].Delay in seeking medical attention and repeated attempts at self‐extraction can cause mucosal damage, edema, and muscle spasms, potentially causing the foreign body to move further upward, leading to complicated surgical interventions 20]. In our case, the patient, intending to evade judicial punishment, did not disclose rectal insertion of ampoules but eventually sought emergency care after experiencing rectal pain and discomfort over 24 h after the ampoule was inserted. Neither did he mention the expulsion of another ampoule during defecation while the operating room was being prepared. This failure to communicate led to a transanal exploration under spinal anesthesia. A similar case by Boucher et al. [21], which involved drug packing and the hiding of truth, led to small bowel obstruction requiring surgical exploration. Our case parallels this scenario where withholding critical information about ampoules led to transanal exploration, which could have been avoided with truthful closure. Also, in cases where drug‐containing foreign bodies are suspected, the use of similar pharmacological agents may confound clinical assessment and increase the risk of toxicity if leakage occurs. Alternative nonopioid analgesics may be preferable in such scenarios.

In conclusion, rectal insertion for concealment of unprotected illicit drug ampoules during apprehension remains an uncommon but challenging clinical scenario requiring high suspicion, thorough history‐taking, and timely imaging for diagnosis. This case emphasizes the importance of accurate history, early imaging, and risk‐based management in rectal foreign bodies. Delayed presentation and lack of truthful disclosure can complicate management, underscoring the importance of patient education and sensitive interrogation techniques. A multidisciplinary approach with surgical backup remains crucial to ensure safe and effective management while minimizing complications.

## Funding

The authors received no funding for this manuscript.

## Ethics Statement

The study is exempt from ethical approval in our institution and with the consent of the patient.

## Consent

Written informed consent was obtained from the patient for publication and any accompanying images. A copy of the written consent is available for review by the editor‐in‐chief of this journal on request.

## Conflicts of Interest

The authors declare no conflicts of interest.

## Data Availability

The data that support the findings of this study are available upon request to the corresponding author. The data are not publicly available due to privacy or ethical restriction.
